# Under-Reporting of Known HIV-Positive Status Among People Living with HIV: A Systematic Review and Meta-analysis

**DOI:** 10.1007/s10461-021-03310-z

**Published:** 2021-05-28

**Authors:** Nirali Soni, Katia Giguère, Marie-Claude Boily, Jessica M. Fogel, Mathieu Maheu-Giroux, Dobromir Dimitrov, Susan H. Eshleman, Kate M. Mitchell

**Affiliations:** 1 Medical Research Council Centre for Global Infectious Disease Analysis, School of Public Health, Imperial College London, London, UK; 2 HIV Prevention Trials Network Modelling Centre, Imperial College London, London, UK; 3 Department of Epidemiology, Biostatistics and Occupational Health, School of Global and Population Health, McGill University, Montreal, Canada; 4 Centre de Recherche du CHUM, Université de Montréal, Montreal, QC Canada; 5 Department of Pathology, Johns Hopkins University School of Medicine, Baltimore, USA; 6 Fred Hutchinson Cancer Research Center, Seattle, USA; 7 Imperial College London, St Mary’s Campus, Praed Street, London, W2 1PG UK

**Keywords:** HIV status, Under-report, Knowledge, Proxy, Bias

## Abstract

Monitoring progress towards the UNAIDS ‘first 90’ target requires accurate estimates of levels of diagnosis among people living with HIV (PLHIV), which is often estimated using self-report. We conducted a systematic review and meta-analysis quantifying under-reporting of known HIV-positive status using objective knowledge proxies. Databases were searched for studies providing self-reported and biological/clinical markers of prior knowledge of HIV-positive status among PLHIV. Random-effects models were used to derive pooled estimates of levels of under-reporting. Thirty-two estimates from 26 studies were included (41,465 PLHIV). The pooled proportion under-reporting known HIV-positive status was 20% (95% confidence interval 13–26%, I^2^ = 99%). In sub-group analysis, under-reporting was higher among men who have sex with men (32%, number of estimates [N_e_] = 10) compared to the general population (9%, N_e_ = 10) and among Black (18%, N_e_ = 5) than non-Black (3%, N_e_ = 3) individuals. Supplementing self-reported data with biological/clinical proxies may improve the validity of the ‘first 90’ estimates.

**Supplementary Information:**

The online version contains supplementary material available at 10.1007/s10461-021-03310-z.

## Introduction

With an estimated 38 million people living with HIV in 2018, ending the HIV/AIDS epidemic remains a key health priority globally [1]. In 2014, UNAIDS introduced the 90-90-90 and 95-95-95 targets with the objective of ending the epidemic by 2030 [2]. The aim was that by 2020, 90% of all people living with HIV (PLHIV) would know their HIV status, 90% of PLHIV who knew their status would be on antiretroviral therapy (ART), and 90% of PLHIV on ART would be virally suppressed, increasing to 95-95-95 by 2030 [2, 3]. Awareness of HIV-positive status—measured in the first UNAIDS target—is necessary to start ART and subsequently become virally suppressed, which increases life expectancy and prevents risk of onwards sexual transmission [4, 5].

Awareness of HIV-positive status is often estimated in surveys as the proportion of all those testing positive who self-report being HIV-positive (e.g. answer that their last HIV test was positive) prior to receiving their test result [6, 7] or from available population data from surveillance systems [8]. The use of self-reported data is convenient and cost-effective, and therefore routinely used in HIV research to measure HIV status knowledge [6, 7], but its quality and validity has been questioned, particularly when involving sensitive information [9, 10]. Recent evidence comparing self-reported data on knowledge of HIV-positive status with biological or clinical markers, such as the presence of antiretroviral (ARV) drugs in the blood, viral load suppression (VLS) and linked medical records, suggests that many PLHIV with prior knowledge of their HIV-positive status do not disclose it, leading to underestimated levels of knowledge of status [11–13] [by almost 20% in one study in the United States (US)] [12], which can misdirect the response.

Given the importance of accurately estimating knowledge of HIV status, we conducted a systematic review and meta-analysis to quantify the level of under-reporting of knowledge of HIV-positive status and identify factors associated with under-reporting.

## Methods

### Search Strategy

We searched MEDLINE, EMBASE, Web of Science, Global Health, and Scopus databases for articles published between January 1st 1985 and October 24th 2019 using terms related to HIV, infection status, self-report, method of prior knowledge of status assessment, and knowledge of status or accuracy domains (Table S1). In addition, we searched *The International AIDS Society* (IAS) [14] conference proceedings from 2017 to 2019 (Table S1).

We also included population-based HIV impact assessment (PHIA) [15] surveys for which full reports with relevant datasets were available by country. Bibliographies of included studies were searched for additional relevant studies.

### Eligibility Criteria

We included publications that assessed knowledge of HIV status by means of self-report among PLHIV with laboratory-confirmed infection, plus at least one of the following methods: ARV drug testing, VLS, medical records, or previous surveys (i.e. PLHIV received HIV-positive test results in a previous study). We excluded reviews and case reports. We did not exclude publications based on language or location.

### Study Selection

We removed duplicate publications, screened by title and abstract for relevance, and then screened potentially relevant full texts for eligibility criteria.

### Data Extraction

From eligible publications and using a standardised form, we directly extracted the primary outcome of interest—proportion of PLHIV under-reporting knowledge of HIV-positive status, if provided. Otherwise, we extracted data on the total number of PLHIV reporting being HIV-positive (A), not reporting HIV-positive status (either reporting unknown or HIV-negative status) but having evidence of prior knowledge of HIV-positive status (e.g. having ARV drugs detected) (B), and the total number of PLHIV with knowledge of HIV-positive status (i.e. C = A + B) and derived the proportion of PLHIV under-reporting knowledge of HIV-positive status (as B/C) (Table S2).

We extracted available estimates or relevant data for the primary outcome for the overall sample and stratified by study site, population type, race, and sex. Where results from the same study were reported in multiple publications, we extracted data from the most recent publication.

We contacted five study authors to get additional data to calculate the outcome of interest, of whom four replied and two provided supplemental data [16–18]. We also included the demographic and health survey (DHS) [19] from Mozambique, which measured our outcome of interest. PHIA and DHS datasets were requested through their respective websites.

Two reviewers (NS and KG) independently performed all stages of screening, selection, and extraction of data, and discrepancies were resolved by KMM.

### Study Quality

We modified the Newcastle–Ottawa scale (NOS) adapted for cross-sectional studies [20] to assess the quality of the included studies, scored on a scale of four stars (Supplementary material, p3).

### Data Analysis

Pooled estimates of the proportion of PLHIV under-reporting knowledge of HIV-positive status and corresponding 95% confidence intervals (CI) were calculated using a random-effects model, using the Sidik–Jonkman method with Hartung–Knapp modification [21, 22] and the double-arcsine transformation [23]. Heterogeneity across studies was assessed using the I^2^ statistic [24].

Where studies provided multiple estimates based on different methods of determining prior knowledge of HIV-positive status, we included only one estimate from that study in the overall pooled estimate, preferentially choosing the estimate expected to be most accurate, starting with medical records (e.g. tested HIV-positive, expected to include PLHIV on ART regardless of viral suppression status, as well as PLHIV diagnosed but not on ART), followed by ARV drug detection, ARV drug detection plus VLS, and VLS alone (which may include undiagnosed viremic controllers). Estimates from multiple geographical locations in a single study were calculated and presented separately, if data was available.

Sub-group and sensitivity analyses, and meta-regressions were conducted to investigate potential sources of heterogeneity due to participant (e.g. population type, sex, country, region, continent, race, legality of homosexuality in country) and study characteristics (e.g. study year, methods of determining prior knowledge of status, interview and sampling methods, study design, quality score (see study quality section)). Study year and quality score were also investigated as continuous variables. R-squared (R^2^) was calculated to determine what proportion of variance could be explained by each factor [24]. We also looked for an association of levels of under-reporting with within-study and country-level ART coverage.

We tested the influence of each individual study estimate on the pooled estimate by conducting a leave-one-out sensitivity analysis (i.e. omitting each study estimate one by one to identify if any estimate has a large effect on the pooled outcome).

For the subset of studies reporting stratified results by race, method of assessing prior knowledge, and sex, we assessed within-study differences in under-reporting of knowledge of HIV-positive status by deriving ratios of the proportion under-reporting for each factor within each study and pooling these ratios across studies.

We also explored if and how the absolute or relative magnitude of reporting bias (i.e. difference between levels of self-reported knowledge and levels of true knowledge of HIV-positive status) varied by levels of self-reported knowledge (% of all PLHIV who self-report HIV-positive status) overall and by population type.

Analyses were carried out using R version 3.6.1 “meta” and “metafor” packages.

### Publication Bias

We assessed publication bias across all included studies using funnel plots and Egger’s test for symmetry [25]. We used the trim-and-fill method to identify potentially missing study estimates [26].

This review was reported in accordance with *Preferred Reporting Items for Systematic Reviews and Meta-Analyses* (PRISMA) guidance (Table S3).

### Role of the Funding Source

The study sponsors had no role in study design, data collection, analysis or interpretation, the writing of the report or the decision to submit the paper for publication.

## Results

### Search Results

**Fig. 1:**
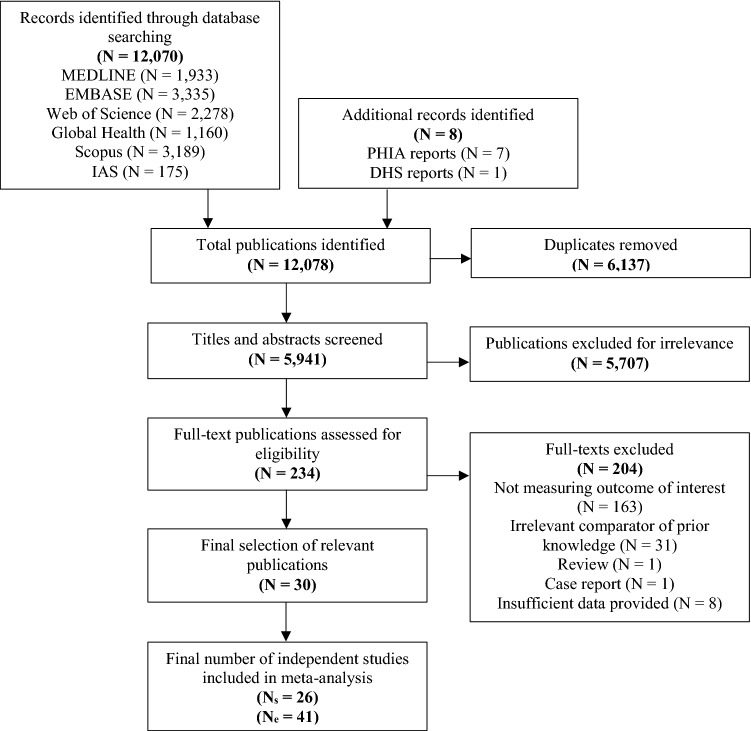
PRISMA flowchart showing the screening and selection process. *DHS* demographic and health survey, *IAS* International AIDS society, *N*_*e*_ number of estimates, *N*_*s*_ number of studies, *PHIA* population-based impact assessment

We identified a total of 12,070 publications, of which 6137 duplicates were removed and 5941 were screened. Of these, 234 full-text publications were assessed for eligibility. A total of 30 eligible publications reporting on 26 independent studies (N_s_) and providing a total of 41 study estimates (N_e_)—some publications reported separate estimates for different populations, study sites or method of assessing prior knowledge of status—were included (Fig. 1).

### Study Characteristics

Key characteristics of included studies are summarised in Table 1. Studies were conducted between 1987 and 2017 but the majority (N_s_ = 23, 85%) were conducted after 2008. They were mainly cross-sectional studies (N_s_ = 20), and otherwise clinical trials (N_s_ = 2) or longitudinal studies (N_s_ = 4). Studies were largely from Africa and North America, representing a total of 41,465 PLHIV, among the general population (N_e_ = 10) or men who have sex with men (MSM; N_e_ = 10), people who inject drugs (PWID; N_e_ = 6), prison inmates (N_e_ = 1), children/adolescents (N_e_ = 1), transgender women (TGW; N_e_ = 2), and female sex workers (FSW; N_e_ = 2). Sample size varied between 15 and 23,474 PLHIV across study estimates.

**Table 1:** Summary of all studies included in the meta-analysis (N_s_ = 26)

First author (Publication date)	Study year	Publication type	Study design	Country	Population type	Number of PLHIV included	Age	Comparator method	Sampling method	Interview type	Quality score
*Africa*											
Hladik (2016) [27]	2012–2013	Journal article	Cross-sectional	Uganda	MSM	79	≥ 18	VLS^a^	RDS	Self-administered	2
Kim (2016) [28]	2012–2013	Journal article	Cross-sectional	Kenya	General population	599	15–64	ARV	2-stage cluster sampling	FTFI	3
Rohr (2017) [29–31]	2014–2015	Journal article	Cohort (analysis on baseline data)	South Africa	General population	1048	≥ 40	ARV	Randomised-sample	FTFI	3
Simms (2017) [32]	2013–2015	Journal article	Cross-sectional	Zimbabwe	Children/adolescents	66	8–17	ARV	Randomised-sample	FTFI	2
Doshi (2018) [16]	2012	Journal article	Cross-sectional	Uganda	FSW	341	15–49	VLS^a^	RDS	Self-administered	2
Fogel (2019) [33, 34]	2015–2016	Journal article	Cohort (analysis on baseline data)	Kenya; Malawi; South Africa	M/TW SM	183	18–44	ARV	Convenience sample	FTFI	3
Hakim (2018) [18]	2014–2015	Journal article	Cross-sectional	Mali	MSM	79	≥ 18	VLS^a^	RDS	FTFI	3
Mooney (2018) [35]	2014	Journal article	Cross-sectional	South Africa	General population (male and female)	317 (109, 208)	18–49	ARV	2-stage cluster sampling	FTFI	3
MPHIA (2018) [36]	2015–2016	Report	Cross-sectional	Malawi	General population (male and female)	2202 (707, 1495)	≥ 15	ARV, VLS^a^	2-stage cluster sampling	FTFI	2
THIS (2018) [37]	2016–2017	Report	Cross-sectional	Tanzania	General population (male and female)	1816 (561, 1255)	≥ 15	ARV, VLS^a^	2-stage cluster sampling	FTFI	2
DHS Mozambique (2019) [38]	2015	Report	Cross-sectional	Mozambique	General population (male and female)	1162, 471	15–59	ARV, VLS^a^	2-stage cluster sampling	FTFI	3
SHIMS2 (2019) [39]	2016–2017	Report	Cross-sectional	Swaziland	General population (male and female)	2997 (972, 2025)	≥ 15	ARV, VLS^a^	2-stage cluster sampling	FTFI	2
ZAMPHIA (2019) [40]	2015–2016	Report	Cross-sectional	Zambia	General population (male and female)	1196 (2438, 770)	≥ 15	ARV, VLS^a^	2-stage cluster sampling	FTFI	2
*Oceania*											
Hakim (2019) [17]	2016	Journal article	Cross-sectional	Papua New Guinea	M/TW SM, FSW	15, 15, 94	> 12	VLS^a^	RDS	FTFI	2
*North America*											
McCusker (1992) [41]	1987–1989	Journal article	Cross-sectional	USA	PWID	38	–	Previous survey	Convenience sample	FTFI	0
Latkin (1998) [42]	1991–1994	Journal article	Cohort (analysis on baseline data)	USA	PWID	104	≥ 18	Previous survey	Convenience sample	FTFI	2
Marzinke (2014) [11]	2009–2011	Journal article	Trial (analysis on baseline data)	USA	Black MSM	340	–	ARV + VLS^a^, VLS^a^	Convenience sample	Self-administered	3
Bai (2014) [43]	2010–2013	Journal article	Cross-sectional	USA	Inmates (male and female)	43 (7, 36)	≥ 16	Medical records	Venue-based sampling	FTFI	2
Madera (2014) [44]	2009–2013	Conference abstract	Cross-sectional	USA	General population	135	–	Medical records	Convenience sample	FTFI	2
Sanchez (2014) [12]	2010–2012	Journal article	Cohort	USA	MSM	237	18–39	ARV, Medical records, VLS^a^	Venue-based and convenience sample	Self-administered	3
An (2016) [45]	2012–2013	Journal article	Cross-sectional	USA	General population (male and female)	498 (336, 162)	≥ 18	Medical records	Venue-based sampling	Self-administered	3
German (2016) [46]	2008	Conference abstract	Cross-sectional	USA	MSM	147	≥ 18	ARV	Venue-based sampling	FTFI	2
German (2017) [47]	2011, 2012, 2014	Conference abstract	Cross-sectional	USA	MSM, PWID	175, 132	≥ 18	ARV	Venue-based sampling (MSM), RDS (PWID)	FTFI	2
Stenger (2018) [48]	2015–2017	Conference abstract	Cross-sectional	USA	MSM	23,474	–	Medical records	Convenience sample	FTFI	1
Hoots (2019) [49, 50]	2014	Journal article	Cross-sectional	USA	MSM	1818	≥ 18	ARV, VLS^b^	Venue-based sampling	FTFI	2
*Multiple locations*											
Fogel (2019) [51]	2015–2016	Journal article	Trial (analysis on baseline data)	Indonesia; Ukraine; Vietnam	PWID	482	18–60	ARV	Convenience sample	FTFI	3

*ARV* antiretroviral, *DHS* demographic and health survey, *FSW* female sex workers, *FTFI* face-to-face interview, *MPHIA* Malawi population-based HIV impact assessment, *MSM* men who have sex with men, *M/TW SM* men and transgender women who have sex with men, *PLHIV* people living with HIV, *PWID* people who inject drugs, *RDS* respondent-driven sampling, *SHIMS2* Swaziland HIV incidence measurement survey 2, *THIS* Tanzania HIV impact survey, *USA* United States of America, *VLS* viral load suppression, *ZAMPHIA* Zambia population-based HIV impact assessment, *P* poor quality (score 0–1), *M* medium quality (score 2–3), *G* good quality (score 4)

^a^Viral suppression defined as < 1000 copies/mL

^b^Viral suppression defined as < 893 copies/mL

Most studies used ARV drug testing (N_e_ = 19), medical records (including surveillance data; N_e_ = 5) or VLS (N_e_ = 14) to determine prior knowledge. Otherwise knowledge was determined using previous surveys (N_e_ = 2), and detection of ARV drugs plus VLS (N_e_ = 1). All but one study used a VLS cut-off of < 1000 copies/mL, which used a cut-off of < 893 copies/mL [49]. Details of the ARV drugs tested for are given in Table S4.

The quality was deemed good, medium, and poor for zero, 24, and two studies, respectively. The most common reason for studies not receiving top quality scores was poor or non-reported response rate (details in Table S5).

### Meta-analysis—Between Studies

**Fig. 2:**
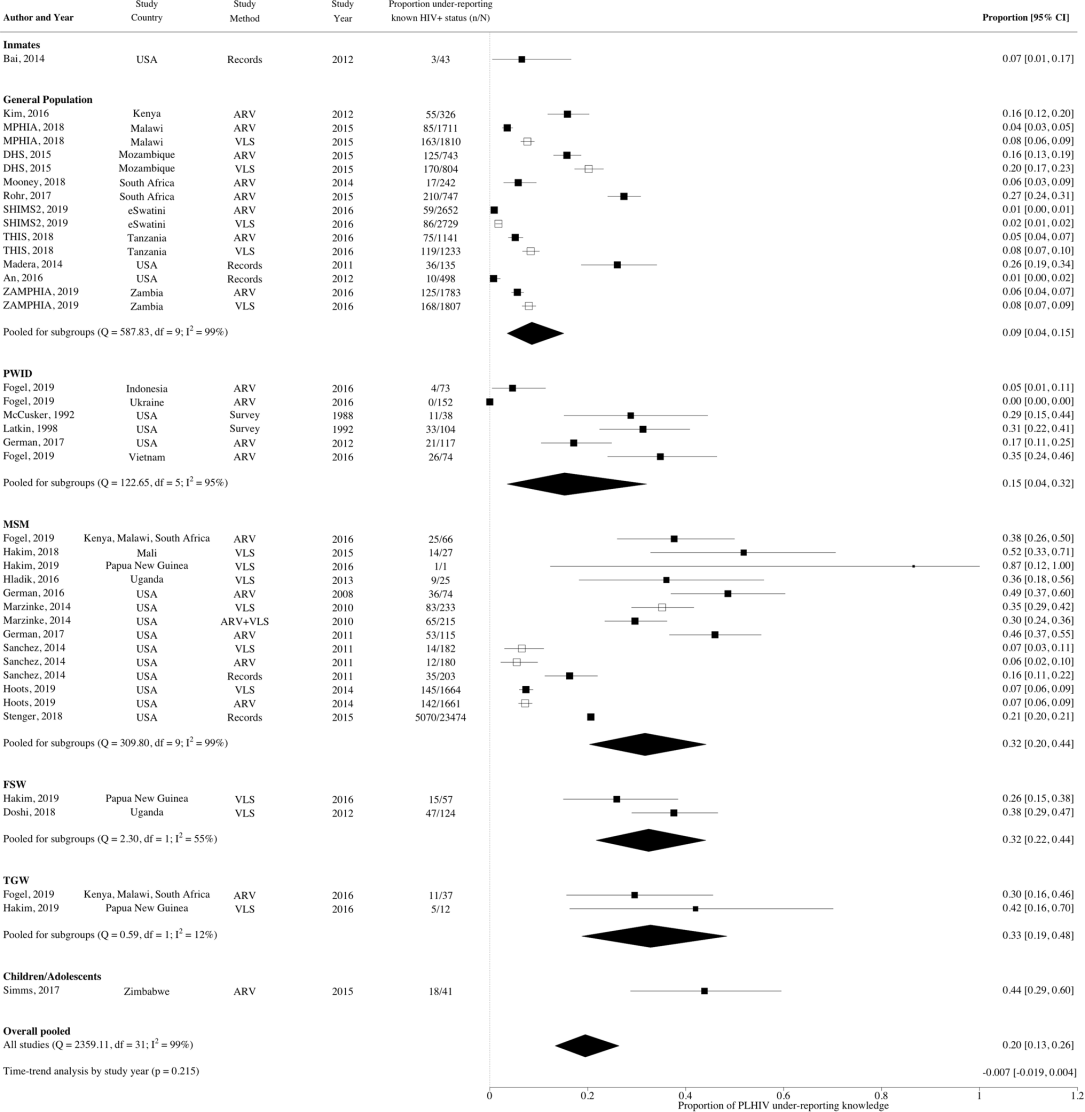
Forest plot showing proportion of people living with HIV under-reporting known HIV-positive status by population type. White squares identify estimates that were excluded from the pooled estimates to avoid counting same population twice. *ARV* antiretroviral, *DHS* demographic and health survey, *FSW* female sex workers, *MPHIA* Malawi population-based HIV impact assessment, *MSM* men who have sex with men, *PLHIV* people living with HIV, *PWID* people who inject drugs, *RE* random effects, *SHIMS2* Swaziland HIV incidence measurement survey 2, *THIS* Tanzania HIV impact survey, *TGW* transgender women, *USA* United States of America, *VLS* viral load suppression, *ZAMPHIA* Zambia population-based HIV impact assessment. Viral suppression considered as < 1000 copies/mL for all but one study which was defined as < 893 copies/mL

**Fig. 3:**
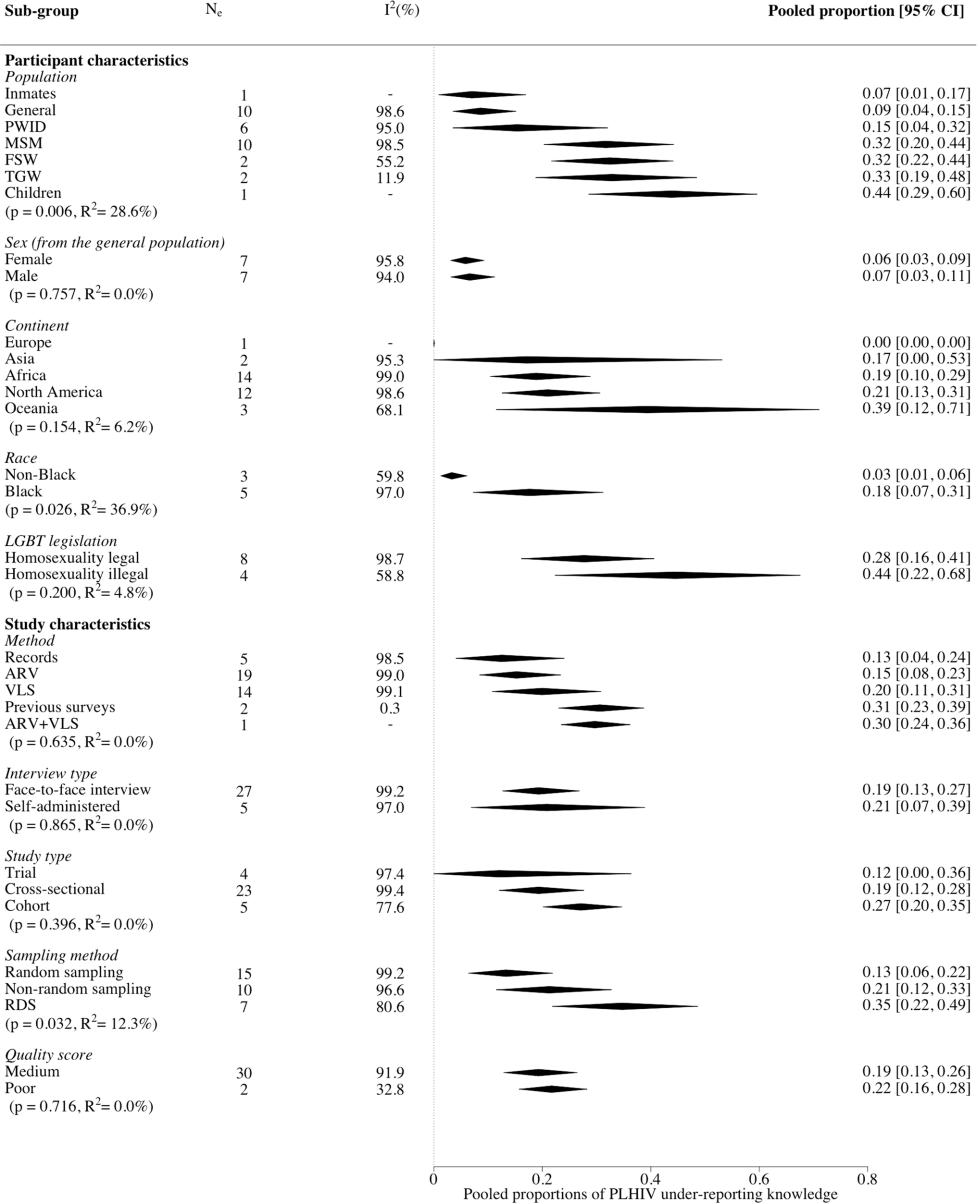
Forest plot showing all sub-group analysis pooled estimates of people living with HIV under-reporting of known HIV-positive status. Sex sub-group only contains studies in the general population. *MSM* men who have sex with men, *PWID* people who inject drugs, *FSW* female sex workers, *TGW* transgender women, *LGBT* lesbian, gay, bisexual, and transgender, *RDS* respondent driven sampling

The overall pooled proportion of PLHIV under-reporting knowledge of HIV-positive status was 20%, (95% CI 13–26%, N_e_ = 32, I^2^ = 99.1%) (Fig. 2) ranging from 1 to 87%. A substantial fraction of the heterogeneity across study estimates could be explained by population type (R^2^ = 29%, N_e_ = 32, p < 0.001) and race (R^2^ = 37%, N_e_ = 8, p < 0.001) (Fig. 2). Higher levels of under-reporting were observed for MSM (32%, 95% CI 20–44%, N_e_ = 10), FSW (32%, 95% CI 22–44%, N_e_ = 2), TGW (33%, 95% CI 19–48%, N_e_ = 2), and children/adolescents (44%, 95% CI 29–60%, N_e_ = 1) compared to the general population (9%, 95% CI 4–15%, N_e_ = 10) (Fig. 3), and for Black than non-Black PLHIV (18% vs. 3% Figs. 3, S1) in the subset of North American studies reporting results by race. Higher levels of under-reporting by MSM than the general population were also observed separately for African and North American studies (Fig. S2). However, no statistically significant differences in under-reporting were observed by region, either overall or by study population (Figs. 3, S2). Within Africa, no statistically significant differences in levels of under-reporting were found between the regions of Eastern Africa (17%, 95% CI 6–33%, N_e_ = 5), Southern Africa (14%, 95% CI 5–28%, N_e_ = 6) and Western Africa (52%, 95% CI 33–70%, N_e_ = 1; z = 1.21, p = 0.227; Fig. S3). However, there was a statistically significant difference observed by African country (z = 2.26, p = 0.024), but not when stratified by population type [general population (z = 0.11, p = 0.915), key populations (z = 1.37, p = 0.172; Figs. S2, S3)]. Levels of under-reporting were not strongly correlated with within-study or country-level ART coverage (Table S6, Fig. S4).

For factors assessing study characteristics, pooled estimates differed by sampling method, where under-reporting was higher for studies using respondent-driven sampling (RDS; 35%, 95% CI 22–49%; N_e_ = 7) and non-random sampling (e.g. convenience sampling; 21%, 95% CI 12–33%; N_e_ = 10) than random sampling methods (e.g. venue-based sampling; 13%, 95% CI 6–22%; N_e_ = 15) (Fig. 3). No statistically significant differences were observed by method of determining prior knowledge or any other participant or study characteristics, or by overall quality score (Figs. 3, S5, S6).

### Meta-analysis—Within-Study Comparisons

Seven studies compared and found lower proportions of under-reporting using ARV drug testing vs. VLS among all participants to determine prior knowledge of status (pooled ratio 0.75, 95% CI 0.64–0.88; Table 2, Fig. S7). With regards to medical records, only one study compared and found lower proportions of under-reporting using ARV drug testing (ratio 0.39, 95% CI 0.21–0.72) and VLS (ratio 0.45, 95% CI 0.25–0.80) vs. medical records. Another study found no substantial difference using ARV drug testing plus VLS vs. VLS alone (ratio 0.85, 95% CI 0.65–1.11) (Table 2, Fig. S7).

**Table 2:** Results for within-study comparisons

Study characteristic	N_e_	Pooled estimate of ratio of proportion [95% CI]	z	p value	I^2^ (%)
Method					
ARV vs VLS	7	0.75 [0.64–0.88]	− 3.5743	0.0004	56.7
ARV vs medical records	1	0.39 [0.21–0.72]	− 2.9836	0.0028	–
VLS vs medical records	1	0.45 [0.25–0.80]	− 2.6966	0.0070	–
ARV + VLS vs VLS	1	0.85 [0.65–1.11]	− 1.2065	0.2276	–
Racial differences					
Non-Black vs Black	3	0.38 [0.17–0.85]	− 2.3448	0.0190	42.7
Sex					
Female vs male	7	0.81 [0.64–1.02]	− 1.7663	0.0773	32.0

*ARV* antiretrovirals drug testing, *N*_*e*_ number of estimates, *VLS* viral load suppression

The pooled ratio of proportion of under-reporting among non-Black PLHIV compared with Black PLHIV in the three studies available was 0.38 (95% CI 0.17–0.85, I^2^ = 43%) and among female PLHIV compared with male PLHIV in the seven general population studies available was 0.81 (95% CI 0.64–1.02, I^2^ = 32%) (Table 2, Figs. S8, S9).

### Regression Analysis for Reporting Bias

Interestingly, the absolute bias did not vary by increasing levels of self-reported knowledge of HIV-positive status overall or by subgroups (Fig. S10a). However, positive associations were observed with the relative bias overall (R^2^ = 0.67, t = 7.23, p < 0.001), for MSM (R^2^ = 0.72, t = 4.67, p = 0.002), and for general populations (R^2^ = 0.52, t = 2.91, p = 0.027) (Fig. S10b).

### Publication Bias

The pooled estimate remained stable in leave-one-out analysis (Fig. S11). The result for the Egger’s test was statistically significant (t = 3.89, p < 0.001), suggesting possible publication bias. The trim and fill analysis found three study estimates likely to be missing from the left-hand side of the funnel plot (Fig. S12). Adding these points would give a pooled proportion of 17% (95% CI 12–25% N_e_ = 35), and a non-significant Egger’s test (z = 1.23, p = 0.22).

## Discussion

We found evidence of under-reporting of knowledge of HIV-positive status being widely prevalent across most studies resulting in substantial underestimation (by 20% overall) of levels of knowledge of HIV-positive status when using self-report alone. The level of under-reporting was more pronounced among key populations such as MSM living with HIV (~ six times more frequent compared to men in the general population) and among Black PLHIV in the US.

Levels of under-reporting of status knowledge were found to be similar between Africa and North America. The majority of studies in the general population were set in Africa and most MSM studies in North America. Stratifying by population type, we found no important differences between the two regions, or between African countries. However, levels of under-reporting were larger for MSM than the general population overall, in Africa and to a lesser extent in North America (where 69% of US PLHIV in 2018 were MSM) [52]. This could be due to structural factors such as differing LGBT legislation and perception, although significant differences in under-reporting were not found between countries where homosexuality was illegal and legal. The lack of an association of under-reporting levels with time suggests no evidence of a decline in stigma, despite efforts to reduce it for PLHIV.

Our sub-group analysis across studies and within-study analysis both highlighted differences by race suggesting greater under-reporting among Black PLHIV. Two studies comparing self-reported results with other data sources (medical records and ARV drug detection) suggested that level of under-reporting rather than awareness differs by race, with Black PLHIV reporting less despite being aware [12, 49]. In the US, Black MSM have a higher HIV prevalence than MSM of other races. This was previously attributed to lower levels of awareness of HIV-positive status among Black MSM [53, 54] but since studies used self-report to determine awareness [54], this theory may need reconsideration [54].

We found differences in under-reporting by sampling method, with significantly higher levels of under-reporting in studies using RDS (note that RDS weights were not accounted for). However, this difference needs to be interpreted with caution as most of the studies using RDS studied MSM—who were more likely to under-report—and there were not enough studies to disentangle population effects from sampling method.

We found little difference in under-reporting by study quality. Many studies scored poorly on the quality scale because they did not give details of non-respondents, which could have introduced methodological bias.

Of all the included studies, only one performed qualitative interviews (among 10 participants from Africa) to investigate possible reasons for under-reporting [35]. In this study, reasons were split into intentional under-reporting from fear of stigma and social ramifications, and unintentional under-reporting from misclassification and misunderstanding of questions [35].

Social desirability bias is thought to contribute to intentionally inaccurate self-reported data, where study participants tend to give socially acceptable responses [42, 55]. The authors of some of the included articles suggested there may be a lack of participant trust from fears of breached confidentiality and leaked information [11, 49]. This could explain the increased under-reporting among key populations like MSM compared to the general population, as well as the racial differences, where these communities may be less trusting of study officials or face more stigma [56–58]. Studies enrolling partners may find increased under-reporting if participants have not previously disclosed their status to their partner [51]. The authors of some of the included articles suggested that under-reporting of HIV-positive status may be due to a belief that participation in the study is limited to HIV-negative individuals or that study enrolment was capped/not allowed for those on ART [11, 34]. This would be particularly relevant for cohort studies and trials, but no statistically significant difference was found by study design in our analysis.

Considering the role of social desirability bias and trust in under-reporting, we expected under-reporting to be lower with self-administered interviews (including audio computer-assisted self-interview) compared to face-to-face, but no differences in under-reporting by interview type were found. This may be because an interviewer could better explain questions, eliminating any misunderstandings arising [35, 49].

The main unintentional errors identified by Mooney et al. were confusion with terminology and problems with temporal questions including recall bias [35]. When asked about “last HIV test results”, some previously diagnosed individuals mistook this for their last CD4 + count or viral load result, inadvertently mis-reporting their status [35]. Misclassification and data entry errors could also lead to apparent misreporting [35, 42].

The absolute magnitude of reporting bias (the difference between self-reported and ‘true’ knowledge of status) was independent of the level of self-reporting, which also meant that relative reporting bias decreased as self-reporting increased. This could provide a correction factor where knowledge of status has been measured using self-reported data alone. Since there was substantial heterogeneity between studies, however, such a correction factor should be applied with caution.

### Limitations of the Comparator Methods

The proxies of knowledge of HIV-positive status used in this study (ARV drug detection, VLS, medical records, and previous surveys) provide more objective measures of knowledge of status than self-report alone, by diminishing the biases discussed above, but do not necessarily have perfect sensitivity or specificity to detect true status knowledge.

There were some indications that ARV drug testing gave significantly smaller estimates of under-reporting compared to VLS from the seven studies directly comparing both methods of determining prior knowledge of status in the same study and in sub-group analysis, albeit not statistically significantly. This differed from our initial accuracy assumption—we expected ARV drug detection to have higher sensitivity—and could indicate lower specificity of VLS.

The presence of ARV drugs in blood could be explained by pre-exposure prophylaxis or recreational ARV use among seroconverted individuals not aware of their HIV status, potentially leading to overestimation of levels of under-reporting. However, it is more likely that using this method underestimates levels of under-reporting since not all PLHIV with knowledge of status have initiated treatment. Furthermore, some ARV drugs may not have been included in assays used for analysis [34] or have short half-lives [28], non-or semi-adherence to complicated regimens may reduce the likelihood of detection, and many studies only looked for specific ARV drugs consistent with standard regimens.

Using VLS as a proxy for knowledge of status could overestimate under-reporting due to inappropriate inclusion of viraemic controllers, who control their viral load without ARV drugs and may not be aware of their HIV status. In some settings, these persons constitute fewer than 1% of PLHIV [59, 60], although, higher frequencies of viraemic controllers have been reported elsewhere [61, 62]. Conversely, this method could underestimate the level of under-reporting since not all PLHIV with knowledge of status may have started taking ARV drugs, may not have taken them for long enough to achieve viral suppression, or may be failing ART.

Only one US study directly compared biomarkers with medical records within the same study, finding that medical records gave higher estimates of levels of under-reporting compared to ARV drug testing or VLS [12] (contrary to our sub-group analysis results). Medical records are likely to have greater sensitivity for identifying PLHIV under-reporting known HIV-positive status compared to using biomarkers—which are likely to give lower bound estimates—since medical records should include everyone with an HIV diagnosis, regardless of whether they started treatment or were virally suppressed on ART. However, medical records could also underestimate under-reporting, as individuals could have had a previous HIV diagnosis without a clear record [30], records may be unavailable due to confidentiality issues, or PLHIV may have tested anonymously or using home rapid test kits [45].

### Strengths and Limitations of Study

To our knowledge, this study is the first to comprehensively assess under-reporting of knowledge of HIV-positive status using objective proxies, including over 41,000 PLHIV. We had enough estimates to investigate differences by region, gender, and race. Our regression analysis of relative reporting bias suggested a potential correction factor for ‘true’ knowledge of status where self-report alone is used.

Considerable heterogeneity remained after sub-group analysis was done, meaning there may be additional factors to those we could assess that could explain heterogeneity, although no one study influenced the pooled estimate. Publication bias was detected towards higher under-reporting, meaning our estimate could be overestimated. On the other hand, our estimate of levels of under-reporting may be underestimated as our proxies of ‘true’ levels of knowledge are more likely to underestimate than overestimate it. There could have been confounding bias in subgroup meta-analyses. However, analysis of within-study comparisons allowed us to identify potential sources of confounding.

## Conclusion and Implications

We found that one in five PLHIV with evidence of prior knowledge of their status did not self-report being HIV-positive. Levels of misreporting were even more pronounced among MSM (one out of three), especially in Africa. In the US, one out of six Black PLHIV, who are disproportionately affected by HIV, did not self-report a previously known HIV-positive status, compared to one out of fifty non-Black PLHIV. These results likely point to differences in structural factors resulting from stigma and repressive environments, which need to be better understood. Further research into the reasons for under-reporting of HIV-positive status is required. Although the biological markers explored in this study do not provide ‘true’ knowledge, they may provide more accurate levels of knowledge than self-report alone and should be used to supplement and/or adjust self-reported data where possible.

## Supplementary Information

Below is the link to the electronic supplementary material.

Supplementary file1 (DOCX 8691 kb)

## Data Availability

All data used in this study is presented in the forest plots.
