# Supplementary material for: Under‑Reporting of Known HIV‑Positive Status Among People Living with HIV: A Systematic Review and Meta‑analysis
Source: AIDS Behav. Author manuscript; Available in PMC 2021 Dec 1. (PMC8602233; doi:10.1007/s10461-021-03310-z)
Supplement: 1738930_Sup_material [file NIHMS1738930-supplement-1738930_Sup_material.docx]

**Supplementary material**

**Under-reporting of known HIV-positive status among people living with HIV: a systematic review and meta-analysis**

Nirali Soni et al.

**Table S1 Search strategy**

| **Database** | **Search terms** |
| --- | --- |
| Ovid MEDLINE(R) ALL | ((Acquired Immunodeficiency Syndrome/ or HIV/ or HIV.mp. or acquired immunodeficiency syndrome.mp. or human immunodeficiency virus.mp. or AIDS.mp. or HIV1.mp. or HIV2.mp. or HIV-1.mp. or HIV-2.mp. or human immunedeficiency virus.mp. or human immuno-deficiency virus.mp. or human immune-deficiency virus.mp. or acquired immunedeficiency syndrome.mp. or acquired immuno-deficiency syndrome.mp. or acquired immune-deficiency syndrome.mp.) **AND** (serostatus or status or seropositiv* or infection or infected).mp. **AND** (self-report* or patient-report* or under-report* or underreport* or selfreport* or disclos* or misreport* or nondisclos* or non-disclos* or undisclosed or stated).mp. **AND** (Viral Load/ or viral load.mp. or virus titer.mp. or Anti-Retroviral Agents/ or anti-retroviral agents.mp. or antiretroviral*.mp. or ARV.mp. or Medical Records/ or medical records.mp. or surveillance report.mp. or inaccura*.mp. or accura*.mp. or valid*.mp. or invalid*.mp. or (verif* or retest* or testing history).mp.) **AND** (know* or knew or aware* or diagnos* or undiagnos* or recognized or recognised or unrecognised or unrecognized or (accura* or inaccura* or reliab* or estimat* or underestimat* or under-estimat* or valid* or invalid* or bias or agree* or discrepan* or consisten* or inconsistent* or verif* or sensitiv*)).mp.) **NOT** (Review.ti. or Case report.mp.) |
| Web of Science (Core collection) | (TS=(“Acquired Immunodeficiency Syndrome” or HIV or “human immunodeficiency virus” or AIDS or HIV1 or HIV2 or HIV-1 or HIV-2 or “human immunedeficiency virus” or “human immuno-deficiency virus” or “human immune-deficiency virus” or “acquired immunedeficiency syndrome” or “acquired immuno-deficiency syndrome” or “acquired immune-deficiency syndrome”) **AND** TS=(serostatus or status or seropositiv* or infection or infected) **AND** TS=(self-report* or patient-report* or under-report* or underreport* or selfreport* or *disclos* or misreport* or stated) **AND** (TS=(“viral load” or “virus titer” or “Anti-Retroviral Agents” or antiretroviral* or ARV or “medical records” or “surveillance report” or *accura* or *valid*) or TS=(verif* or retest* or “testing history”)) **AND** (TS=(know* or knew or aware* or *diagnos* or *recognized or *recognised) or TS=(*accura* or reliab* or *estimat* or *valid* or bias or agree* or discrepan* or *consisten* or verif* or sensitiv*)) **AND** WC=(infectious diseases) **NOT** (TI=(Review) or TS=(“Case report” or animal*))) |
| EMBASE (classic + embase) | ((Acquired Immunodeficiency Syndrome or HIV or human immunodeficiency virus or AIDS or HIV1 or HIV2 or HIV-1 or HIV-2 or human immunedeficiency virus or human immuno-deficiency virus or human immune-deficiency virus or acquired immunedeficiency syndrome or acquired immuno-deficiency syndrome or acquired immune-deficiency syndrome) **AND** (serostatus or status or seropositiv* or infection or infected) **AND** (self-report* or patient-report* or under-report* or underreport* or selfreport* or disclos* or misreport* or nondisclos* or non-disclos* or undisclosed or stated) **AND** (viral load or virus titer or anti-retroviral agents or antiretroviral* or ARV or medical records or surveillance report or inaccura* or accura* or valid* or invalid* or (verif* or retest* or testing history)) **AND** (know* or knew or aware* or diagnos* or undiagnos* or recognized or recognised or unrecognised or unrecognized or (accura* or inaccura* or reliab* or estimat* or underestimat* or under-estimat* or valid* or invalid* or bias or agree* or discrepan* or consisten* or inconsistent* or verif* or sensitiv*))).mp. **NOT** (Review.ti. or Case report.mp.) |
| Global Health (OVID) | ((Acquired Immunodeficiency Syndrome or HIV or human immunodeficiency virus or AIDS or HIV1 or HIV2 or HIV-1 or HIV-2 or human immunedeficiency virus or human immuno-deficiency virus or human immune-deficiency virus or acquired immunedeficiency syndrome or acquired immuno-deficiency syndrome or acquired immune-deficiency syndrome) **AND** (serostatus or status or seropositiv* or infection or infected) **AND** (self-report* or patient-report* or under-report* or underreport* or selfreport* or disclos* or misreport* or nondisclos* or non-disclos* or undisclosed or stated) **AND** (viral load or virus titer or anti-retroviral agents or antiretroviral* or ARV or medical records or surveillance report or inaccura* or accura* or valid* or invalid* or (verif* or retest* or testing history)) **AND** (know* or knew or aware* or diagnos* or undiagnos* or recognized or recognised or unrecognised or unrecognized or (accura* or inaccura* or reliab* or estimat* or underestimat* or under-estimat* or valid* or invalid* or bias or agree* or discrepan* or consisten* or inconsistent* or verif* or sensitiv*))).mp. **NOT** (Review.ti. or Case report.mp.) |
| Scopus | ( ( TITLE-ABS-KEY ( "Acquired Immunodeficiency Syndrome"  or  hiv  or  "human immunodeficiency virus"  or  aids  or  hiv1  or  hiv2  or  hiv-1  or  hiv-2  OR  "human immunedeficiency virus"  or  "human immuno-deficiency virus"  or  "human immune-deficiency virus"  or  "acquired immunedeficiency syndrome"  or  "acquired immuno-deficiency syndrome"  or  "acquired immune-deficiency syndrome" ) )  **AND**  ( TITLE-ABS-KEY ( serostatus  or  status  or  seropositiv*  or  infection  or  infected ) )  **AND**  ( TITLE-ABS-KEY ( self-report*  or  patient-report*  or  under-report*  or  underreport*  or  selfreport*  or  $disclos*  or  stated ) )  **AND**  ( TITLE-ABS-KEY ("viral load"  or  "virus titer"  or  "anti-retroviral agents"  or  antiretroviral*  or  arv  or  "medical records"  or  "surveillance report"  or  *accura*  or  *valid* )  or  TITLE-ABS-KEY ( verif*  or  retest*  or  "testing history" ) )  **AND**  ( TITLE-ABS-KEY ( know*  or  knew  or  aware*  or  *diagnos*  or  *recognized  or  *recognised )  or TITLE-ABS-KEY ( *accura*  or  reliab*  or  *estimat*  or  *valid*  or  bias  or  agree*  or  discrepan*  or  *consisten*  or  verif*  or  sensitiv* ) ) )  **AND NOT**  ( TITLE-ABS-KEY ( "case report")  or  TITLE ( review ) ) |
| International AIDS Society conference abstracts | Know, aware, diagnos, accura, valid, reliab, self-report, diclos |

**Table S2 Outcome of interest calculation**

|  | **Self-report positive** | **Don’t self-report positive** | **Total** |
| --- | --- | --- | --- |
| Aware* | A Number of PLHIV who self-report being HIV-positive | B Number of PLHIV with undisclosed HIV-positive status | C Total number of PLHIV aware of HIV-positive status |

*According to our gold standard: ARV drug detection, VLS, medical records, or previous surveys.

**Adapted Newcastle-Ottawa scale**

**Selection:** (Maximum 2 stars (*))

1) Representativeness of the sample:

a) Truly representative of the average in the target population. *

*(All subjects selected from the population or random sampling methods used e.g. cluster randomisation)*

b) Somewhat representative of the average in the target population. *

*(Non-random sampling methods may be used e.g. convenience sampling or unweighted RDS, but sample representative of target population)*

c) Selected group of users. *(All selected participants are from a single population, which is not representative of the target population e.g. MSM selected from a sexual health clinic)*

d) No description of the sampling strategy.

2) Non-respondents:

a) Comparability between respondents and non-respondents’ characteristics is established, and the response rate is satisfactory (>60%). *

*(Comparison of non-responders and responders done)*

b) The response rate is unsatisfactory (<60%).

c) The response rate is reported but the comparability between respondents and non-respondents is unsatisfactory.

d) No description of the response rate.

e) The response rate is reported but no description of the characteristics of the responders and the non-responders.

**Outcome:** (Maximum 2 stars)

1) Ascertainment of self-reported status:

a) Questions asked to find out status provided. *

b) No description of questions.

2) Assessment of prior knowledge:

a) Uses surveillance data for knowledge. *

b) Uses biological confirmation of knowledge. *

c) Uses previous studies.

**Table S3 PRISMA checklist**

| Section/topic 12 | # | Checklist item | Reported on page # |
| --- | --- | --- | --- |
| **TITLE** | | | |
| Title | 1 | Identify the report as a systematic review, meta-analysis, or both. | 1 |
| **ABSTRACT** | | | |
| Structured summary | 2 | Provide a structured summary including, as applicable: background; objectives; data sources; study eligibility criteria, participants, and interventions; study appraisal and synthesis methods; results; limitations; conclusions and implications of key findings; systematic review registration number. | 3 |
| **INTRODUCTION** | | | |
| Rationale | 3 | Describe the rationale for the review in the context of what is already known. | 3 |
| Objectives + aims | 4 | Provide an explicit statement of questions being addressed with reference to participants, interventions, comparisons, outcomes, and study design (PICOS). | 4 |
| **METHODS** | | | |
| Protocol and registration | 5 | Indicate if a review protocol exists, if and where it can be accessed (e.g., Web address), and, if available, provide registration information including registration number. | No review protocol exists |
| Eligibility criteria | 6 | Specify study characteristics (e.g., PICOS, length of follow-up) and report characteristics (e.g., years considered, language, publication status) used as criteria for eligibility, giving rationale. | 5 |
| Information sources | 7 | Describe all information sources (e.g., databases with dates of coverage, contact with study authors to identify additional studies) in the search and date last searched. | 4 |
| Search | 8 | Present full electronic search strategy for at least one database, including any limits used, such that it could be repeated. | 4 |
| Study selection | 9 | State the process for selecting studies (i.e., screening, eligibility, included in systematic review, and, if applicable, included in the meta-analysis). | 5 |
| Data collection process | 10 | Describe method of data extraction from reports (e.g., piloted forms, independently, in duplicate) and any processes for obtaining and confirming data from investigators. | 5 |
| Data items | 11 | List and define all variables for which data were sought (e.g., PICOS, funding sources) and any assumptions and simplifications made. | 6 |
| Risk of bias in individual studies | 12 | Describe methods used for assessing risk of bias of individual studies (including specification of whether this was done at the study or outcome level), and how this information is to be used in any data synthesis. | 6 |
| Summary measures | 13 | State the principal summary measures (e.g., risk ratio, difference in means). | 5 |
| Synthesis of results | 14 | Describe the methods of handling data and combining results of studies, if done, including measures of consistency (e.g., I^2^) for each meta-analysis. | 6 |
| Risk of bias across studies | 15 | Specify any assessment of risk of bias that may affect the cumulative evidence (e.g., publication bias, selective reporting within studies). | 7 |
| Additional analyses | 16 | Describe methods of additional analyses (e.g., sensitivity or subgroup analyses, meta-regression), if done, indicating which were pre-specified. | 6 |
| RESULTS | | | |
| Study selection | 17 | Give numbers of studies screened, assessed for eligibility, and included in the review, with reasons for exclusions at each stage, ideally with a flow diagram. | 8 |
| Study characteristics | 18 | For each study, present characteristics for which data were extracted (e.g., study size, PICOS, follow-up period) and provide the citations. | 8 |
| Risk of bias within studies | 19 | Present data on risk of bias of each study and, if available, any outcome-level assessment (see Item 12). | 8 |
| Results of individual studies | 20 | For all outcomes considered (benefits or harms), present, for each study: (a) simple summary data for each intervention group and (b) effect estimates and confidence intervals, ideally with a forest plot. | 8 |
| Synthesis of results | 21 | Present results of each meta-analysis done, including confidence intervals and measures of consistency. | 9 |
| Risk of bias across studies | 22 | Present results of any assessment of risk of bias across studies (see Item 15). | 10 |
| Additional analysis | 23 | Give results of additional analyses, if done (e.g., sensitivity or subgroup analyses, meta-regression [see Item 16]). | 9 |
| DISCUSSION | | | |
| Summary of evidence | 24 | Summarize the main findings including the strength of evidence for each main outcome; consider their relevance to key groups (e.g., health care providers, users, and policy makers). | 10 |
| Limitations | 25 | Discuss limitations at study and outcome level (e.g., risk of bias), and at review level (e.g., incomplete retrieval of identified research, reporting bias). | 14 |
| Conclusions | 26 | Provide a general interpretation of the results in the context of other evidence, and implications for future research. | 15 |
| FUNDING | | | |
| Funding | 27 | Describe sources of funding for the systematic review and other support (e.g., supply of data); role of funders for the systematic review. | 2 |

**Table S4 ARV drugs that were tested for**

|  | |  |  |
| --- | --- | --- | --- |
| **Author** | **Publication year** | | **ARV drugs tested for** |
| DHS Mozambique^1^ | 2015 | | AZT, 3TC and NVP were selected because they represent the first line ART regime. Respondents were considered to have evidence of ARV drug use if at least one of the three drugs was detected. |
| Fogel^2^ | 2019 | | 20 ARV drugs tested for 3 NNRTIs, 6 NRTIs, 9 PIs and raltegravir and maraviroc using qualitative assay. ART regimens recommended in Indonesia, Ukraine and Vietnam at the time of the study included an NNRTI or PI with 2 NRTIs. Participants were considered to be on ART if an NNRTI or PI was detected, with or without NRTIs. |
| Fogel^3^ | 2019 | | 20 drugs tested for, 6 NRTIs, 3 NNRTIs, 9 PI, an integrase strand transfer inhibitor and a CCR5 receptor antagonist. Detection of one was considered previously diagnosed. |
| German^4^ | 2016 | | 20 drugs tested for – not specified. |
| German^5^ | 2017 | | 20 drugs tested for – not specified. |
| Hoots^6^ | 2019 | | 3TC, raltegravir, abacavir, FTC, TDF, EFV, RTV. Those non reporting being HIV-positive but with at least one ARV detected were classed as misreporting. |
| Kim^7^ | 2016 | | Qualitative ARV assay testing for: NVP, EFV, 3TC and LPV. At the time of the study the first-line standardised ARV regimen in Kenya was TDF + 3TC + EFV or NVP, and second-line regimen was AZT +3TC + lipotenovir/ritonavir. Samples which tested positive for any of these drugs were considered as having ARV biomarkers present. |
| Marzinke^8^ | 2014 | | 15 drugs tested for: 9 PIs (amprenavir, ATV, DRV, IDV, LPV, NFV, RTV, SQV, and tipranavir TPV), 2 NNRTIs (EFV and NVP), and 4 nucleoside reverse transcriptase inhibitors (FTC, 3TC, TDF, and AZT). |
| Mooney^9^ | 2018 | | Qualitative assay testing for: EFV, LPV, NV, to include both first- and second-line regimen. Because TDF and FTC are only given in combination with EFV, they were not included. |
| MPHIA^10^ | 2018 | | EFV, ATV and LPV, were selected as markers for the most commonly prescribed first- and second-line regimens. Samples from participants who had suppressed viral loads and/or self-reported on ART, but had no evidence of the first three compounds, were tested for nevirapine. |
| Rohr^11^ | 2017 | | 3TC or FTC, which are standard drugs in first- and second- line regimens. Presence of either drug was considered positive for ART. |
| Sanchez^12^ | 2014 | | NRTIs (FTC, 3TC, TDF, AZT), NNRTIs (EFV, NVP), PIs (ATV, DRV, fasomprenavir, IDV, LPV, nelfinavir, RTV, SQV and TPV). |
| SHIMS2^13^ | 2019 | | EFV, LPV, and NVP. These ARVs were also selected based on their relatively long half-lives and are common first- and second-line regimens. |
| Simms^14^ | 2017 | | 3TC, TDF, NVP, and RTV. All ART regimens in Zimbabwe following national guidelines included at least 1 of these drugs. |
| THIS^15^ | 2018 | | EFV, LPV, and NVP. These ARVs were also selected based on their relatively long half-lives and are common first- and second-line regimens. |
| ZAMPHIA^16^ | 2019 | | EFV, ATV and LPV, were selected as markers for the most commonly prescribed first- and second-line regimens. Samples from participants who had suppressed viral loads and/or self-reported on ART, but had no evidence of the first three compounds, were tested for nevirapine. |

DHS = demographic and health survey, MPHIA = Malawi population-based HIV impact assessment, SHIMS2 = Swaziland HIV incidence measurement survey 2, THIS = Tanzania HIV impact survey, ZAMPHIA = Zambia population-based HIV impact assessment. 3TC = lamivudine, ATV = atazanavir , AZT = zidovudine, DRV = darunavir , EFV = efavirenz, FTC = emtricitabine, IDV = indinavir, LPV = lopinavir, NFV = nelfinavir, NNRTI = non-nucleoside reverse transcriptase inhibitors, NRTI = nucleoside/nucleotide reverse transcriptase inhibitors, NVP = nevirapine, PI = protease inhibitors, RTV = ritonavir, SQV = saquinavir, TDF = tenofovir, TPV = tipranavir

**Table S5 Newcastle-Ottawa scale results**

|  | |  | Selection (maximum 2) | | | Outcome (maximum 2) | | |  |  | | |
| --- | --- | --- | --- | --- | --- | --- | --- | --- | --- | --- | --- | --- |
| ***Author*** | ***Publication year*** | | ***Representativeness***  ***(Study design)***  *a. Truly representative, b. Somewhat representative, c. Selected group, d. No description* | ***Non-respondents***  *a. Response rate satisfactory and comparability between respondents and non-respondents, b. Response rate unsatisfactory, c. Comparability unsatisfactory, d. No description of response rate, e. no description of comparability* | ***Ascertainment of self-report***  *a. Details of questions provided, b. No description of questions* | | ***Ascertainment of prior knowledge***  *a. Uses surveillance data, b. Uses biological confirmation, c. Uses previous studies* | ***Total*** | | | ***Quality*** |  |
| An^17^ | 2016 | | a(*) | e | a(*) | | a(*) | 3 | | | M |  |
| Bai^18^ | 2014 | | a(*) | e | b | | a(*) | 2 | | | M |  |
| DHS Mozambique^1^ | 2015 | | a(*) | e | a(*) | | b(*) | 3 | | | M |  |
| Doshi^19^ | 2018 | | b(*) | d | b | | b(*) | 2 | | | M |  |
| Fogel^2^ | 2019 | | b(*) | d | a(*) | | b(*) | 3 | | | M |  |
| Fogel^3^ | 2019 | | b(*) | d | a(*) | | b(*) | 3 | | | M |  |
| German^4^ | 2016 | | a(*) | d | b | | b(*) | 2 | | | M |  |
| German^5^ | 2017 | | a(*) | d | b | | b(*) | 2 | | | M |  |
| Hakim^20^ | 2018 | | b(*) | e | a(*) | | b(*) | 3 | | | M |  |
| Hakim^21^ | 2019 | | b(*) | d | b | | b(*) | 2 | | | M |  |
| Hladik^22^ | 2016 | | b(*) | e | b | | b(*) | 2 | | | M |  |
| Hoots^6^ | 2019 | | a(*) | d | b | | b(*) | 2 | | | M |  |
| Kim^7^ | 2016 | | a(*) | d | a(*) | | b(*) | 3 | | | M |  |
| Latkin^23^ | 1998 | | b(*) | d | a(*) | | c | 2 | | | M |  |
| Madera^24^ | 2014 | | d | b | a(*) | | a(*) | 2 | | | M |  |
| Marzinke^8^ | 2014 | | b(*) | e | a(*) | | b(*) | 3 | | | M |  |
| McCusker^25^ | 1992 | | c | e | b | | c | 0 | | | P |  |
| Mooney^9^ | 2018 | | a(*) | e | a(*) | | b(*) | 3 | | | M |  |
| MPHIA^10^ | 2018 | | a(*) | e | b | | b(*) | 2 | | | M |  |
| Rohr^11^ | 2017 | | a(*) | e | a(*) | | b(*) | 3 | | | M |  |
| Sanchez^12^ | 2014 | | a(*) | d | a(*) | | b(*) | 3 | | | M |  |
| SHIMS2^13^ | 2019 | | a(*) | e | b | | b(*) | 2 | | | M |  |
| Simms^14^ | 2017 | | a(*) | e | b | | b(*) | 2 | | | M |  |
| Stenger^26^ | 2018 | | d | d | b | | a(*) | 1 | | | P |  |
| THIS^15^ | 2018 | | a(*) | e | b | | b(*) | 2 | | | M |  |
| ZAMPHIA^16^ | 2019 | | a(*) | e | b | | b(*) | 2 | | | M |  |

* = stars awarded. DHS = demographic and health survey, MPHIA = Malawi population-based HIV impact assessment, SHIMS2 = Swaziland HIV incidence measurement survey 2, THIS = Tanzania HIV impact survey, ZAMPHIA = Zambia population-based HIV impact assessment. P= poor quality (score 0-1), M = medium quality (score 2-3), G = good quality (score 4).

**Table S6 Estimates for within-study and country-level ART coverage**

| ***Author*** | ***Publication year*** | ***Country*** | ***Within-study ART coverage (%)*** | ***Country-level ART coverage (%)*** |
| --- | --- | --- | --- | --- |
| DHS Mozambique^1^ | 2015 | Mozambique | 35 | 60^27^ |
| Fogel^2^ | 2019 | Indonesia | 18 | 12^28^ |
| Fogel^2^ | 2019 | Ukraine | 5 | 54^27^ |
| Fogel^2^ | 2019 | Vietnam | 2 | 70^27^ |
| Fogel^3^ | 2019 | Kenya | 72* | 74^27^ |
| Fogel^3^ | 2019 | Malawi | 22* | 79^27^ |
| Fogel^3^ | 2019 | South Africa | 24* | 70^27^ |
| German^4^ | 2016 | USA | 33* | 70^28^ |
| German^5^ | 2017 | USA | 47* | 70^28^ |
| German^5^ | 2017 | USA | 58* | 70^28^ |
| Hoots^6^ | 2019 | USA | 48* | 70^28^ |
| Kim^7^ | 2016 | Kenya | 43 | 74^27^ |
| Marzinke^8^ | 2014 | USA | 86* | 70^28^ |
| Mooney^9^ | 2018 | South Africa | 52 | 70^27^ |
| MPHIA^10^ | 2018 | Malawi | 91 | 79^27^ |
| Rohr^11^ | 2017 | South Africa | 41 | 70^27^ |
| Sanchez^12^ | 2014 | USA | 20* | 70^28^ |
| SHIMS2^13^ | 2019 | eSwatini | 89 | 95^27^ |
| Simms^14^ | 2017 | Zimbabwe | 55 | 85^27^ |
| THIS^15^ | 2018 | Tanzania | 94 | 75^27^ |
| ZAMPHIA^16^ | 2019 | Zambia | 87 | 85^27^ |

* = studies measuring ART coverage only among those PLHIV who do not self-report being HIV-positive. Within-study ART coverage was estimated from ARV detection.

***Figure S1: Forest plot showing proportion of PLHIV under-reporting known HIV-positive status by race in studies conducted in North America***

***Figure S2: Forest plot comparing under-reporting of known HIV+ status among MSM and the general population by region***

MSM = men who have sex with men, USA = United States of America.

***Figure S3: Forest plot comparing under-reporting of known HIV+ status by African region.*** FSW = female sex workers, MSM = men who have sex with men.


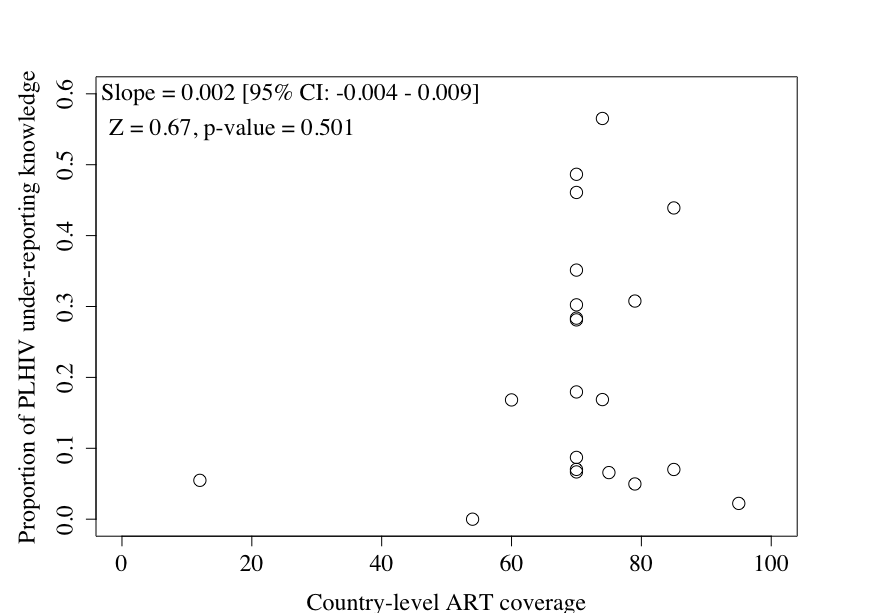

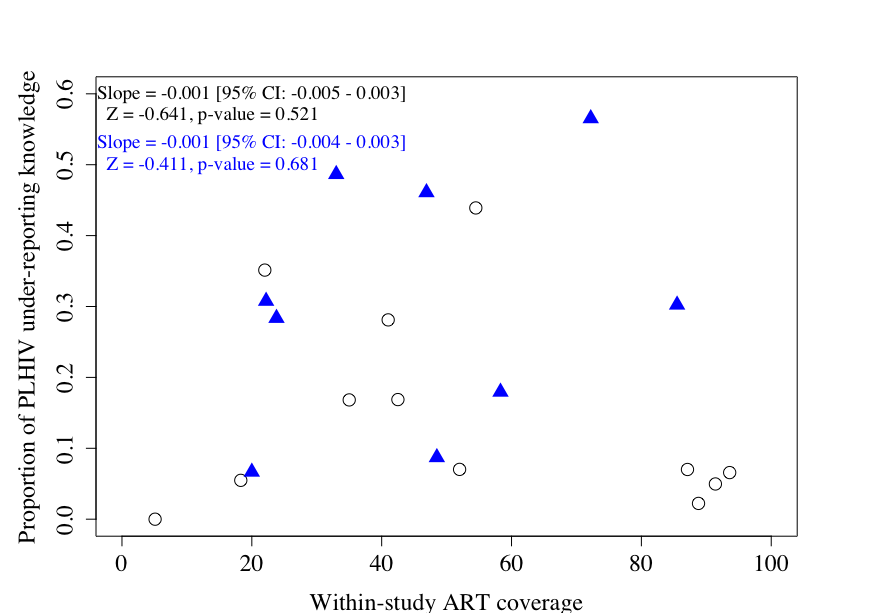


***Figure S4: Association between self-reported knowledge of and (a) within-study ART coverage*** *or* ***(b) county-level ART coverage****. For within-study ART coverage, circles indicate studies measuring ART coverage among all PLHIV. Blue triangles indicate studies measuring ART coverage only among those PLHIV who do not self-report being HIV-positive. Slope estimate in black includes only studies measuring ART coverage among all PLHIV (N_e_=12) and slope estimate in blue include all study estimates (N_e_=21).* Within-study ART coverage was estimated from ARV detection.

b

a


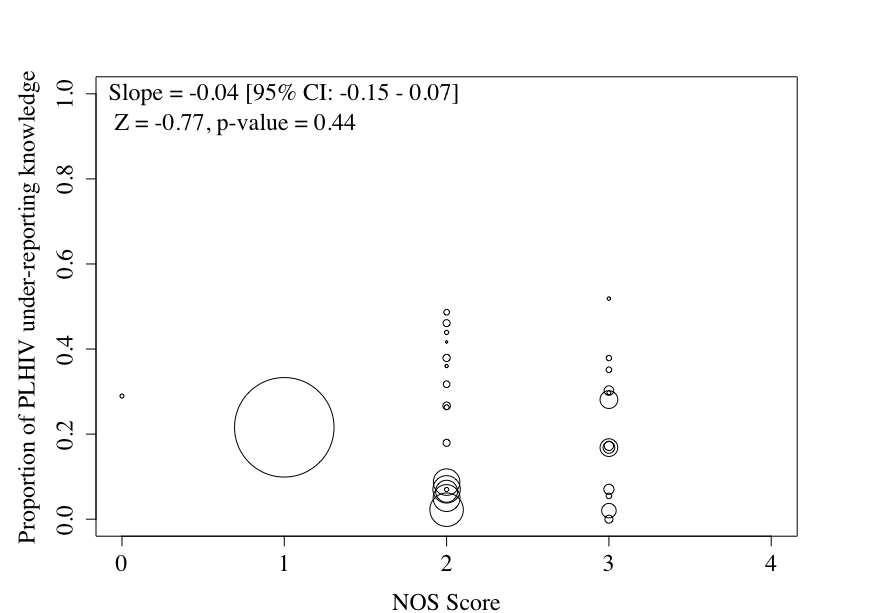


***Figure S5: Scatter plot of the proportion under-reporting known HIV+ status by total quality score***

The diameters of the points are proportional to the inverse variance.


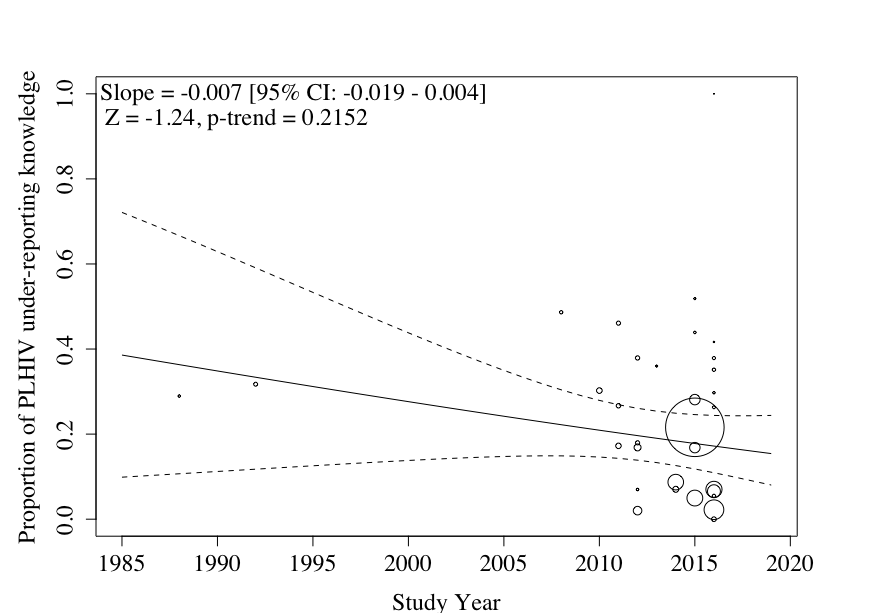


***Figure S6: Time trends in the proportion of people living with under-reporting known HIV+ status***

The diameters of the points are proportional to the inverse variance. Each circle represents a single estimate.

***Figure S7: Forest plot showing the within-study differences in proportion under-reporting by method of determining prior knowledge (******for studies that used more than one method to assess prior knowledge of status***), ARV = antiretroviral drugs, VLS = viral load suppression


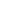

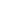

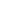

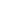

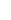

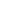

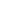

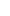

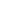

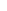

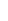


**

***Figure S8: Forest plot showing the within-study differences in under-reporting by race in North America***

***Figure S9: Forest plot showing the within-study differences in under-reporting by sex.*** *Note these estimates come from general population studies only.*

b

a

| **Relative difference** | **β** | **t** | **p-value** | **Adjusted R^2^** |  |
| --- | --- | --- | --- | --- | --- |
| MSM | 0.86 [0.42-1.30] | 4.67 | 0.0023 | 0.7223 |  |
| General population | 0.41 [0.07-0.75] | 2.91 | 0.0269 | 0.5166 |  |
| PWID | 0.39 [-0.42-1.20] | 1.33 | 0.2540 | 0.1337 |  |
| All | 0.75 [0.54-0.96] | 7.23 | <0.0001 | 0.6664 |  |

| **Absolute difference** | **β** | **t** | **p-value** | **Adjusted R^2^** |
| --- | --- | --- | --- | --- |
| MSM | 0.02 [-0.24-0.28] | 0.21 | 0.8436 | -0.1361 |
| General population | 0.20 [-0.09-0.49] | 1.71 | 0.1376 | 0.2164 |
| PWID | 0.02 [-0.85-0.89] | 0.06 | 0.9536 | -0.2488 |
| All | 0.12 [-0.05-0.28] | 1.42 | 0.1682 | 0.0376 |

***Figure S10: Relationship between self-reported knowledge of status by population type and (a) the absolute magnitude of bias*** *(% self-report - % true knowledge) or* ***(b) the relative magnitude of bias*** *((% self-report – % true knowledge) / % true knowledge). Dashed line shows the relationship for all points. Population analysis done for populations with at least 3 separate estimates.*

***Figure S11: Leave-one-out analysis showing the changes in the overall pooled estimate as each estimate is removed one by one.*** *Dashed line shows pooled estimate.*

******

***Figure S12: a) Funnel plot showing the distribution of all of the included study estimates by standard error; b) Trim and fill funnel plot showing the distribution of all included study estimates and missing studies***

b

a

***References:***

1. Survey of indicators on immunization, malaria and HIV/AIDS. Moçambique. <https://dhsprogram.com/pubs/pdf/AIS12/AIS12.pdf> [Accessed: 2^nd^ March 2020]
2. Fogel JM, Zhang Y, Palumbo PJ, Guo X, Clarke W, Breaud A, et al. Use of antiretroviral drug testing to assess the accuracy of self-reported data from HIV-infected people who inject drugs. AIDS and Behavior. 2019;23(8): 2101–2108. doi:10.1007/s10461-018-2379- 8
3. Fogel JM, Zhang Y, Guo X, Clarke W, Breaud A, Cummings V, et al. Reliability of self-reported HIV status among African MSM screened for HPTN 075, In: 25^th^ Conference on Retroviruses and Opportunistic Infections, CROI 2018, Topics in Antiviral Medicine. United States: International Antiviral Society; 2018.
4. German D, Shearer K, Park JN, Flynn C, Latkin C, Laeyendecker O, et al. Factors associated with misreporting HIV status among MSM from Baltimore [CROI abstract 906]. In Special Issue: Abstracts From the 2016 Conference on Retroviruses and Opportunistic Infections. Topics in Antiviral Med. 2016;24(e-1):384.
5. German D, Shearer K, Flynn C, Latkin CA, Laeyendecker O, Quinn T, et al. Examination of unrecognized and misreported HIV status in Baltimore MSM and PWID [CROI Abstract 904]. In Special Issue: Abstracts From the 2017 Conference on Retroviruses and Opportunistic Infections. Topics in Antiviral Medicine. 2017;25(suppl 1):384-385.
6. Hoots BE, Wejnert C, Martin A, Haaland R, Masciotra S, Sionean C, et al. Undisclosed HIV infection among MSM in a behavioral surveillance study. AIDS. 2019;33(5): 913– 918. doi:10.1097/QAD.0000000000002147
7. Kim AA, Mukui I, Young PW, Mirjahangir J, Mwanyumba S, Wamicwe J, et al. Undisclosed HIV infection and antiretroviral therapy use in the Kenya AIDS indicator survey 2012: relevance to national targets for HIV diagnosis and treatment. AIDS. 2016;30(17): 2685–2695. doi:10.1097/QAD.0000000000001227
8. Marzinke MA, Clarke W, Wang L, Cummings V, Liu TY, Piwowar-Manning E, et al. Nondisclosure of HIV status in a clinical trial setting: Antiretroviral drug screening can help distinguish between newly diagnosed and previously diagnosed HIV infection. Clinical Infectious Diseases. 2014; 58 (1): 117-120. doi://dx.doi.org/10.1093/cid/cit672
9. Mooney AC, Campbell CK, Ratlhagana M-J, Grignon JS, Mazibuko S, Agnew E, et al. Beyond social desirability bias: Investigating inconsistencies in self-reported HIV testing and treatment behaviors among HIV-positive adults in North West Province, South Africa. AIDS and Behavior. 2018;22(7): 2368–2379. doi:10.1007/s10461-018-2155-9
10. Malawi Population-based HIV Impact Assessment (MPHIA) 2015-2016. <https://phia.icap.columbia.edu/wp-content/uploads/2019/08/MPHIA-Final-Report_web.pdf> [Accessed: 12^th^ January 2020]
11. Rohr JK, Xavier Gómez-Olivé F, Rosenberg M, Manne-Goehler J, Geldsetzer P, Wagner RG, et al. Performance of self-reported HIV status in determining true HIV status among older adults in rural South Africa: a validation study. Journal of the International AIDS Society. 2017;20(1): 21691. doi:10.7448/IAS.20.1.21691
12. Sanchez TH, Kelley CF, Rosenberg E, Luisi N, O’Hara B, Lambert R, et al. Lack of awareness of Human Immunodeficiency Virus (HIV) infection: Problems and solutions with self-reported HIV serostatus of men who have sex with men. Open Forum Infectious Diseases. 2014;1(2): ofu084. doi:10.1093/ofid/ofu084
13. Swaziland HIV Incident Measurement Survey 2 (SHIMS2) 2016-2017. <https://phia.icap.columbia.edu/wp-content/uploads/2019/05/SHIMS2_Final-Report_05.03.2019_forWEB.pdf> [Accessed: 12^th^ January 2020]
14. Simms V, Dauya E, Dakshina S, Bandason T, et al. Community burden of undiagnosed HIV infection among adolescents in Zimbabwe following primary healthcare-based provider-initiated HIV testing and counselling: A cross-sectional survey. PLOS Medicine. 2017;14 (7), e1002360. doi:10.1371/journal.pmed.1002360.
15. Tanzania HIV Impact Assessment (A Population-based HIV Impact Assessment) THIS 2016-2017. <https://phia.icap.columbia.edu/wp-content/uploads/2019/06/FINAL_THIS-2016-2017_Final-Report__06.21.19_for-web_TS.pdf> [Accessed: 12^th^ January 2020]
16. Zambia Population-based HIV Impact Assessment (ZAMPHIA) 2016 <https://phia.icap.columbia.edu/wp-content/uploads/2019/03/ZAMPHIA-Final-Report__2.26.19.pdf> [Accessed: 10^th^ January 2020]
17. An Q, Chronister K, Song R, Pearson M, Pan Y, Yang B, et al. Comparison of self-reported HIV testing data with medical records data in Houston, TX 2012-2013. Annals of Epidemiology. 2016; 16(4): 255-60. Doi:10.1016/j.annepidem.2016.02.013.
18. Bai J, Mukherjee D, Befus M, Apa Z, et al. Concordance between medical records and interview data in correctional facilities. BMC Medical Research Methodology.2014;14 (1). doi:10.1186/1471-2288-14-50.
19. Doshi R, Sande E, Ogwal M, Kiyingi H, et al. Progress toward UNAIDS 90-90-90 targets: A respondent-driven survey among female sex workers in Kampala, Uganda. PLOS ONE. 2018;13 (9), e0201352. doi:10.1371/journal.pone.0201352.
20. Hakim A, Coy K, Patnaik P, Telly N, et al. An urgent need for HIV testing among men who have sex with men and transgender women in Bamako, Mali: Low awareness of HIV infection and viral suppression among those living with HIV. PLOS ONE. 2018;13 (11), e0207363. doi:10.1371/journal.pone.0207363.
21. Hakim A, Badman S, Weikum D, Amos A, et al. Considerable distance to reach 90-90-90 targets among female sex workers, men who have sex with men and transgender women in Port Moresby, Papua New Guinea: findings from a cross-sectional respondent-driven sampling survey. Sexually Transmitted Infections. 2019;96 (2), 143-150. doi:10.1136/sextrans-2019-053961.
22. Hladik W, Sande E, Berry M, Ganafa S, Kiyingi H, Kusiima J, et al. Men who have sex with men in Kampala, Uganda: Results from a bio-behavioral respondent driven sampling survey. AIDS and Behavior. 2017; 21 (5): 1478-1490. doi: //dx.doi.org/10.1007/s10461-016- 1535-2
23. Latkin CA, Vlahov D. Socially desirable response tendency as a correlate of accuracy of self- reported HIV serostatus for HIV seropositive injection drug users. Addiction. 1998; 93 (8): 1191-1197. doi: 10.1046/j.1360-0443.1998.93811917.x
24. Madera RT. Validating self-reported HIV status from STD surveillance network (SSuN) enhanced gonorrhea interviews, Philadelphia, 2009-2013 [STD Prevention Conference abstract TP134]. Sexually Transmitted Diseases. 2014; 41(suppl 1): S75
25. McCusker J, Stoddard AM, McCarthy E. The validity of self-reported HIV antibody test results. American Journal of Public Health. 1992;82(4): 567–569. doi:10.2105/AJPH.82.4.567
26. Stenger M, Bauer H, Madera R, Pathela P, Schumacher C, Skyphard L, et al. Non-disclosure of HIV status among MSM diagnosed and reported with gonorrhea, indings from the STD surveillance network (SSuN) July 2015 - June 2017. Sexually Transmitted Diseases. 2018;45 (Supplement 2): S100.
27. UNAIDS. AIDSinfo. <https://aidsinfo.unaids.org/> [Accessed: 15^th^ July 2020]
28. Wang H, Wolock TM, Carter A, Nguyen G, Kyu HH, Gakidou E, et al. Estimates of global, regional, and national incidence, prevalence, and mortality of HIV, 1980–2015: the Global Burden of Disease Study 2015. *The Lancet HIV*. [Online] Elsevier; 2016;3(8): e361–e387. doi:[10.1016/S2352-3018(16)30087-X](https://doi.org/10.1016/S2352-3018(16)30087-X)
